# Supplementary material for: Improving Lipophagy by Restoring Rab7 Cycle: Protective Effects of Quercetin on Ethanol-Induced Liver Steatosis
Source: Nutrients. 2022 Feb 4;14(3):658. doi: 10.3390/nu14030658 (PMC8915175; doi:10.3390/nu14030658)
Supplement: Supplementary file 1 [file nutrients-14-00658-s001.zip › nutrients-1516293.pdf]

Supplementary Materials for

Improvement Lipophagy by Restoring Rab7 Cycle: Protective Effects of Quercetin on Ethanol-Induced Liver Steatosis

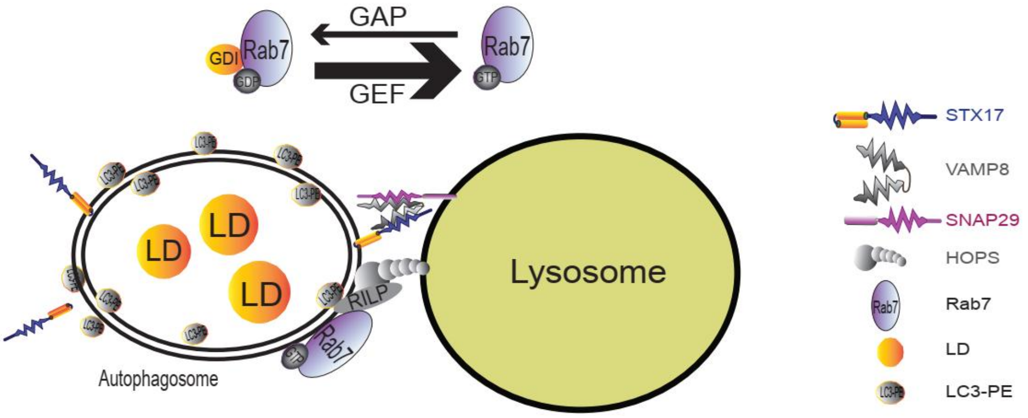

Fig. S1 Schematic diagram of the roles of membrane fusion-associated proteins in the process of autophagosome and lysosome fusion. Rab7 and SNAREs as the main membrane fusion proteins were measured. Rab7 GTPase is efficient for autophagosome-lysosome fusion[1] and SNAREs can pull two opposing membranes for fusion. The Rab GTPases as molecular switches that are activated by GTP binding and inactivated following GTP hydrolysis[2]. GDP-Rab7 bound with GDI as non-activated form exists in cytoplasmic and the GDP-Rab7 is activated to GTP-Rab7 by GEF complex on autophagosomes and then recruit variety effectors, such as RILP, the retromer complex, PLEKHM1 and HOPs. Then the membrane fusion is realized by SNAREs, and syntaxin17 (Stx17), vesicle-associated membrane protein 8 (VAMP8), and synaptosome-associated protein 29 (SNAP29) as three main SNAREs proteins can form a 4-helix bundle. Stx17 as a Qa-SNARE (t-SNARE, localization in mature autophagosomes) can bind with SNAP29 (contain two SNARE domains, Qb and Qc-SNAREs, resides in the cytoplasm and transiently associate with membrane) and further bounding with VAMP8 (R-SNARE, or v-SNARE, localization in lysosomes membrane) to zipper into SNARE bundle[3-5].

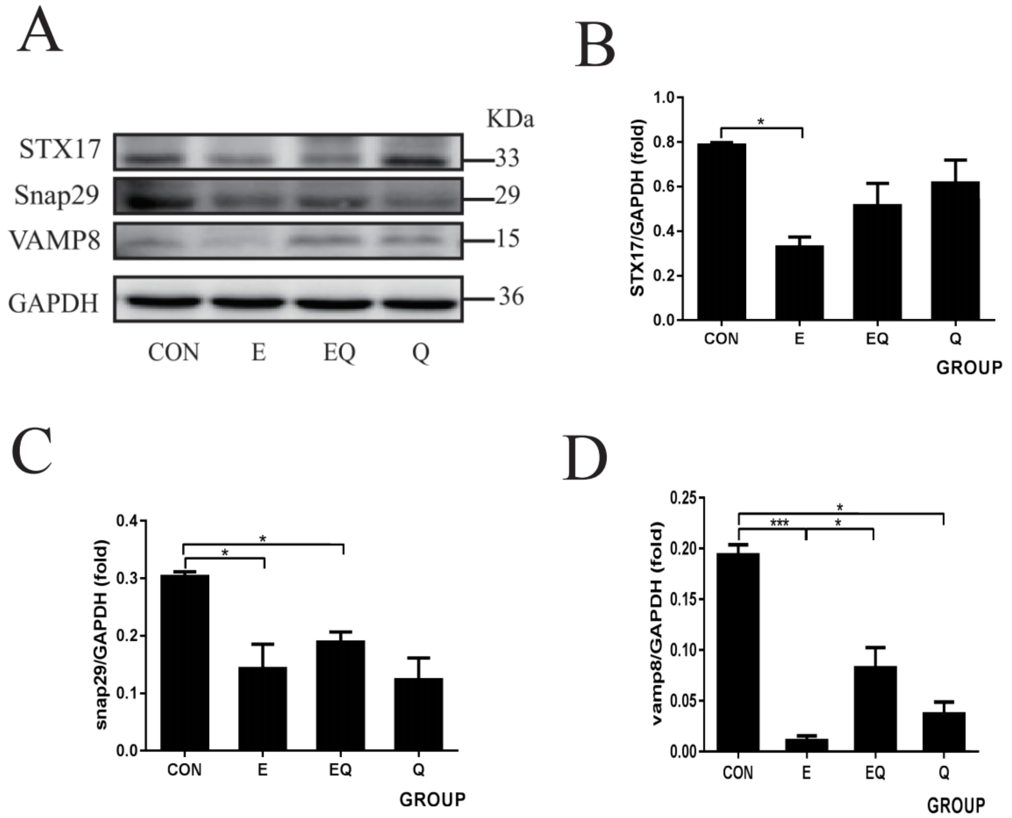

Fig. S2 Effects of quercetin on the expression of SNAREs in mice treated with chronic alcohol. (**a**) Western blot of SNAREs (STX17, Snap29 and VAMP8). (**b-d**) Densitometry analysis of (**a**). The GAPDH was used as a protein loading control, and the results were quantified in three independent experiments per condition. Data shown are means ± SD (n=6). *P < 0.05, **P < 0.01, ***P < 0.001. CON: normal control group; E: ethanol group; EQ: ethanol plus quercetin group; Q: quercetin group.

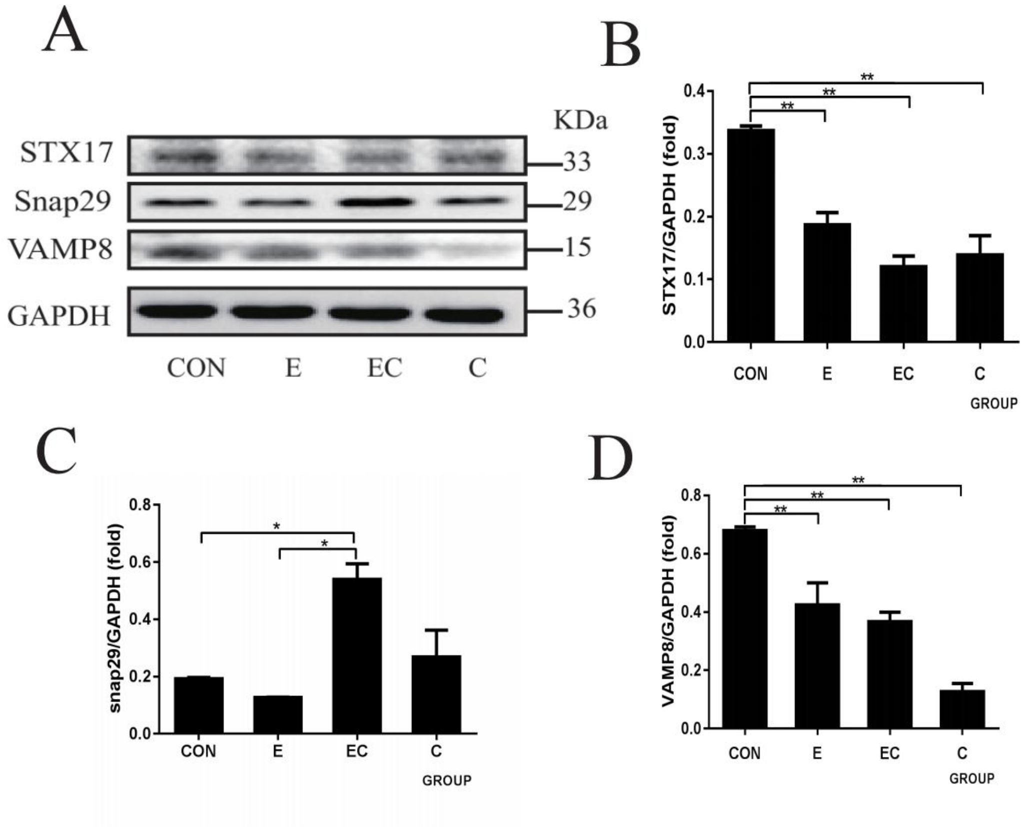

Fig. S3 The expression of CYP2E1 after transfeced with CYP2E1 plasmid and the chang of the expression of SNAREs after treating by RAB7 activation inhibitor CID1067700 in Hepg2 cells. (**a**) Western blot of CYP2E1. (**b**) Western blot of SNAREs (STX17, Snap29 and VAMP8). (**c-e**) Densitometry analysis of (**b**). The GAPDH was used as a protein loading control, and the results were quantified in three independent experiments per condition. Data shown are means ± SD (n=3). *$P < 0.05$, **$P < 0.01$, ***$P < 0.001$. CON: normal control group; E: ethanol group; EC: ethanol plus CID1067700 group; C: CID1067700 group.

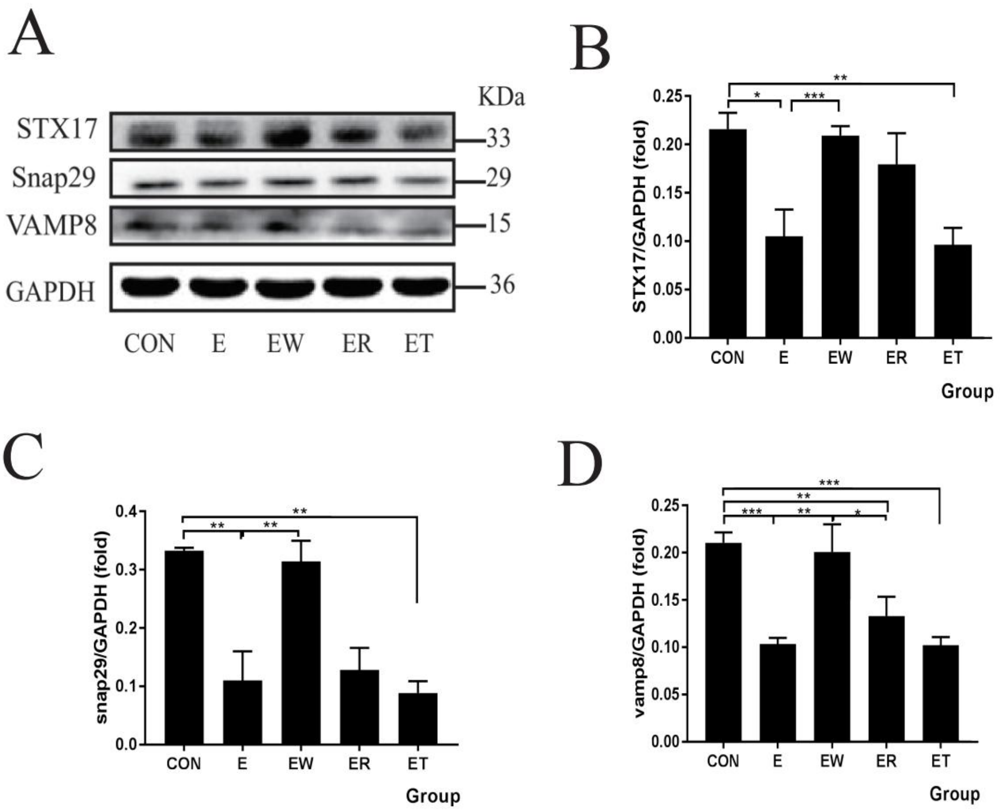

Fig. S4 Effect of different forms Rab7 in SNAREs expression in Hepg2 cells. (a) Western blot of SNAREs (STX17, Snap29 and VAMP8). (**b-d**) Densitometry analysis of (**a**). The GAPDH was used as a protein loading control, and the results were quantified in three independent experiments per condition. Data shown are means ± SD (n=3). **P* < 0.05, ***P* < 0.01, ****P* < 0.001. CON: normal control group; E: ethanol group; EW: Hepg2 cells expressing Rab-Wt plus ethanol; ER: Hepg2 cells expressing Rab-Q67L (active form) plus ethanol; ET: Hepg2 cells expressing Rab-T22N (inactive form) plus ethanol.

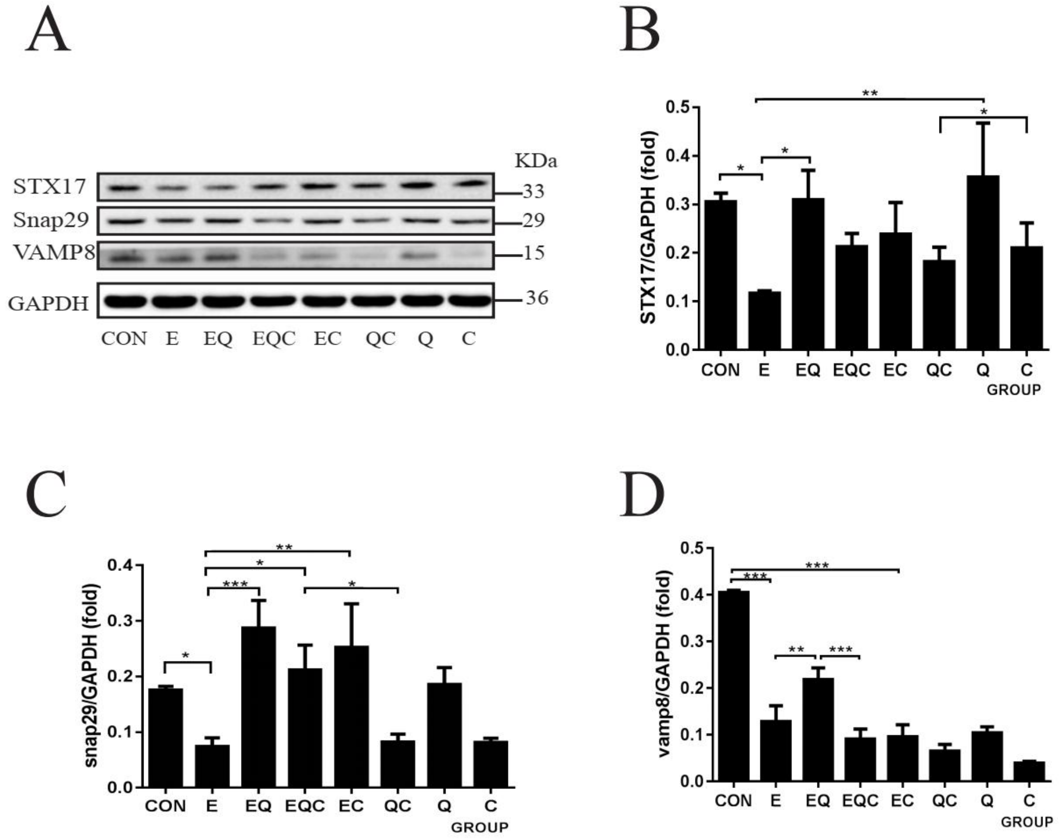

Fig. S5 Effect of quercetin on SNAREs in Hepg2 cells. (a) Western blot of SNAREs (STX17, Snap29 and VAMP8). (**b-d**) Densitometry analysis of (**a**) Data shown are means ± SD (n=3). **P* < 0.05, ***P* < 0.01, ****P* < 0.001. CON: normal control group; E: ethanol group; EQ: ethanol plus quercetin group; EQC: CID1067700 an ethanol plus quercetin group; EC: ethanol plus CID1067700 group; QC: quercetin plus CID1067700 group; C: CID1067700 group Q: quercetin group.

References

1. Takáts, S. *et al.* Interaction of the HOPS complex with Syntaxin 17 mediates autophagosome clearance in Drosophila. *Mol Biol Cell* **25**, 1338-1354 (2014).
2. Kawakami, A. *et al.* Rab7 regulates maturation of melanosomal matrix protein gp100/Pmel17/Silv. *J Invest Dermatol* **128**, 143-150 (2008).
3. Itakura, E., Kishi-Itakura, C. & Mizushima, N. The hairpin-type tail-anchored SNARE syntaxin 17 targets to autophagosomes for fusion with endosomes/lysosomes. *Cell* **151**, 1256-1269 (2012).
4. Morelli, E. *et al.* Multiple functions of the SNARE protein Snap29 in autophagy, endocytic, and exocytic trafficking during epithelial formation in Drosophila. *Autophagy* **10**, 2251-2268 (2014).

5. Spang, A. Cell biology: Bulky tether proteins aid membrane fusion. *Nature* **551**, 576-577 (2017).
